# Effect of Bait Quantity and Trap Color on the Trapping Efficacy of the Pheromone Trap for the Red Palm Weevil, *Rhynchophorus ferrugineus*

**DOI:** 10.1673/031.012.12002

**Published:** 2012-10-20

**Authors:** Abdullah Mohamed Abuagla, Mohammad Ali Al-Deeb

**Affiliations:** ^1^ Research and Development Division, Abu Dhabi Food Control Authority, P.O. Box: 52150, Abu Dhabi, UAE; ^2^ Biology Department, Faculty of Science, United Arab Emirates University, P.O. Box 17551, Al-Ain, UAE

**Keywords:** black trap, date palm, trap attraction, UAE

## Abstract

The red palm weevil, *Rhynchophorus ferrugineus* (Olivier) (Curculionidae: Coleoptera), is not native to the United Arab Emirates (UAE). Since its arrival in 1985, it has been causing major damage to date palm trees. A primary control strategy has been the use of pheromone baited traps. The objectives of this study were to determine the quantity of bait, and the best trap color, to obtain the maximum catch of *R. ferrugineus* under field conditions in the UAE. Traps with 100, 300, or 500 g of dates as bait collected the same number of *R. ferrugineus* adults. Captures in black traps were significantly higher than captures in red, yellow, or white traps. Thus, using a black pheromone trap containing 100 g of dates can significantly enhance *R. ferrugineus* control efforts, and can help considerably in reducing the red palm weevil's deleterious impact on date palm production in UAE.

## Introduction

The red palm weevil, *Rhynchophorus ferrugineus* (Olivier) (Curculionidae: Coleoptera), was first reported in the United Arab Emirates (UAE) in 1985 (El-Ezaby et al. 1998). Since then, it has become the most important insect pest on date palm trees in the country. This pest is not native to UAE, and it was inadvertently introduced into the country with imported infested offshoots. *R. ferrugineus* attacks a broad range of palms in southern Asia (Murphy and Brisco 1999). Because the damaging larval stage is concealed, tree injury is often severe when it is discovered (Abraham et al. 1998). Traps baited with the *R. ferrugineus* aggregation pheromone and date fruits are an effective measure to control *R. ferrugineus* (Abraham et al. 1999; Faleiro 2000). Since the discovery of *R. ferrugineus* in UAE, pest control authorities have used mainly white (and in limited cases yellow) pheromone baited traps. However, no published research work recommended the use of these two trap colors. Other reports indicated that black (Hallett et al. 1999), green (Ajlan and Abdulsalam 2000), and brown-reddish (Sansano et al. 2008) traps collected more *R. ferrugineus* adults than white or yellow traps. In the UAE, a study by Al-Saoud et al. (2010) showed that red traps collected significantly more *R. ferrugineus* compared to the white and yellow traps. They indicated that *R. ferrugineus* adults were more attracted to dark colors. This prompted the investigation of the use of dark colored traps, and the optimization of the quantity of bait. The objectives of the current study were to compare the effect of bait quantity on captures of *R. ferrugineus*, and to compare the effect of trap color on captures of *R. ferrugineus* under field conditions in the UAE. The importance of this study comes from the fact that it marks the commencement of using black traps in the control of *R. ferrugineus* in UAE. Although this color was reported to be attractive to this pest by Hallett et al. (1999), it was not adopted in UAE, most likely because the trap design was different. It was also possible that different *R. ferrugineus* populations might have different responses. In this regard, Salama and Saker (2002) reported finding major differences in DNA fingerprints among morphologically different forms of *R. ferrugineus* collected from date plantations in Egypt. Such genetic differences could have effects on the response of the adults to semiochemicals (Faleiro 2006).

## Materials and Methods

### Study sites

The experiments were conducted at date palm plantations at Al-Ain (24° 16′ N, 55° 36′ E), UAE. Plantations were selected based on two criteria: they contained trees of similar ages, and *R. ferrugineus* infestation levels were nearly the same. The second factor was determined based on data from a one-year pheromone trap. Trees in the plantations were under the same *R. ferrugineus* integrated pest management (IPM) practices in UAE.

### Trap design and installation

**Figure 1.**
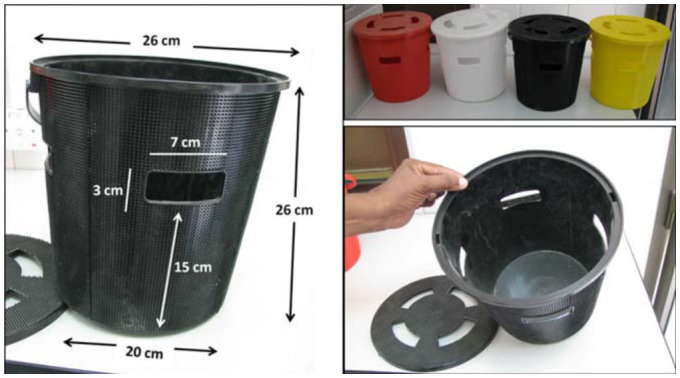
Red palm weevil trap dimensions (left), tested colors (upper right), and trap inside and lid (lower right). High quality figures are available online.

The trap was a 10 L plastic bucket (Figure 1). Trap height was 26 cm, and the diameters of the top and bottom were 26 and 20 cm, respectively. Each trap and its lid had four equidistant rectangular (3 × 7 cm) openings to allow *R. ferrugineus* entrance. The outer surface of the trap was rough with projections (3 mm) to help *R. ferrugineus* climb on and enter the trap. Each trap contained one pheromone dispenser, (P028 Ferrolure+®) containing 700 mg (ChemTica Int., Costa Rica), that was attached to the lower surface of the trap lid by a wire, and replaced monthly. Traps were buried in the ground to the level of the openings. The distance between traps was 70 m, and each trap was 3– 4 m away from the nearest date palm tree. Each trap contained dates as bait, and water as a fermentation medium. The dates and water were changed every 15 days.

**Table 1.**
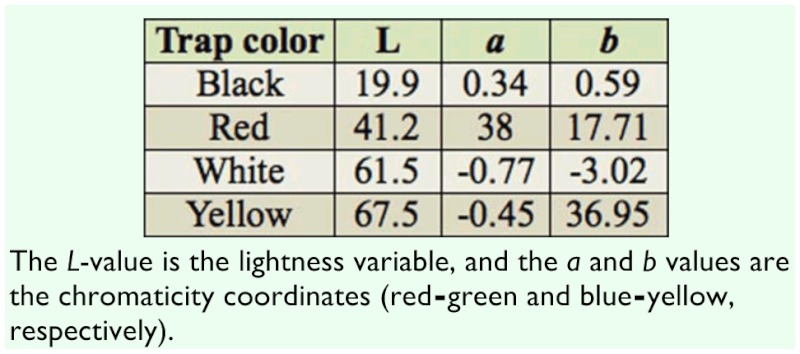
Trap surface color values (L^*^a^*^b) measured by chromameter.

### Bait quantity experiment

A total of 60 white traps were installed in five locations in the field. Each location was a date palm plantation in which four treatments (0, 100, 300, and 500 g of dates per trap) were tested. Each trap contained water as a date fermentation medium. Each treatment was represented by three traps (replications) per location. Captured insects were collected weekly. The experiment was conducted from May 2009 to June 2010.

### Trap color experiment

In the current study, the trap color was the color of the plastic material itself (original color), whereas in Al-Saoud et al. (2010), the traps were white and experimentally sprayed with different paints (painted color). A total of 48 traps were installed in four locations in the field. Each location was a date palm plantation in which four trap colors (white, yellow, red, and black) were tested. Each trap color was represented by three traps (replications) per location. Trap surface color values (L^*^a^*^b) were measured by a chromameter (Table 1). The *L*-value is the lightness variable, and the *a* and *b* values are the chromaticity coordinates (red-green and blue-yellow, respectively). These three values can be used to define a point in three-dimensional space that characterizes a color in absolute terms. Each trap contained water and 100 g of dates. Captured insects were collected weekly. The experiment was conducted from May 2009 to June 2010.

**Figure 2.**
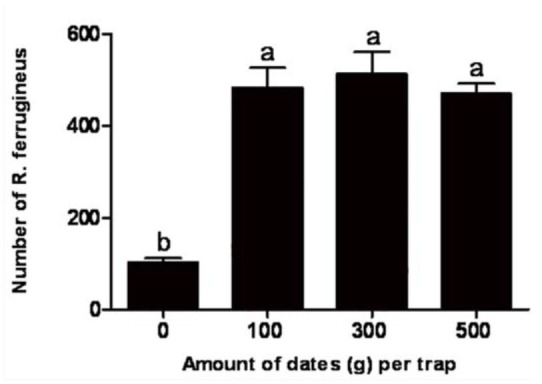
Mean (± SEM) number of *Rhynchophorus ferrugineus* adults per trap captured in pheromone-baited traps containing different quantities of dates. Bars labeled with different letters are significantly different (*p* < 0.05). High quality figures are available online.

### Statistical analysis

The experimental design was a randomized complete block. Trap catch data were subjected to a one-way analysis of variance (ANOVA) using the PROC GLM procedure, and the means were separated by the least significant difference LSD procedure of the SAS statistical software (SAS 2001).

## Results

### Bait quantity.

Significant differences in the mean number of captured *R. ferrugineus* adults occurred between traps containing bait and traps containing water (no bait) (*F* = 34.06; df=3, 18; *p* < 0.0004) (Figure 2). The maximum number of *R. ferrugineus* was collected in traps containing 300 g of dates (512.3), and was not significantly different from the catch in traps containing 100 g (482.7) or 500 g (469.3) (*t* = -0.63, df = 6, *p* < 0.55; *t* = -0.92, df = 6, *p* < 0.3942, respectively). The minimum number of *R. ferrugineus* (103) was collected by the trap containing 0 g of dates, and was significantly different from the traps containing 100, 300, or 500 g of dates (*t* = 8.10, df = 6, *p* < 0.0002; *t* = 8.73, df = 6, *p* < 0.0001; *t* = 7.82, df = 6, *p* < 0.0002, respectively).

### Trap color.

**Figure 3.**
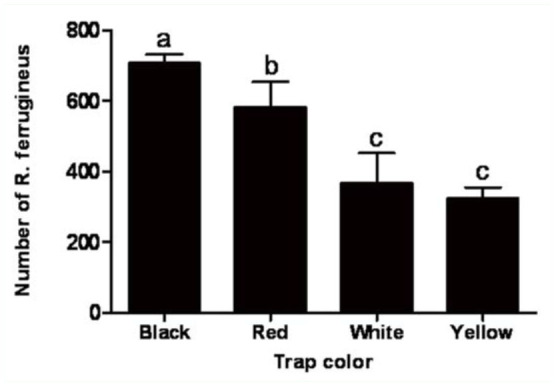
Mean (± SEM) number of *Rhynchophorus ferrugineus* adults per trap captured in pheromone-baited traps of different colors. Bars labeled with different letters are significantly different (*p* < 0.05). High quality figures are available online.

Significant differences in the mean total catch of adult *R. ferrugineus* occurred among the four trap colors tested (*F* = 29.26; df = 3, 18; *p* < 0.0006) (Figure 3). Black traps captured the maximum number (707.0) of *R. ferrugineus*, and the catch in black traps was significantly different from the catch in red, white, and yellow traps (*t* = -2.66, df = 6, *p* < 0.0374; *t* = -7.19, df = 6, *p* < 0.0004; *t* = -8.11, df = 6, *p* < 0.0002, respectively). The red traps captured fewer *R. ferrugineus* (581.3) than black traps, but significantly more than the white (367.3) and yellow (324.3) traps (t = 4.53, df = 6, *p* < 0.004; *t* = -5.44, df = 6, *p* < 0.0016, respectively). The yellow traps caught the least *R. ferrugineus*, but the number was not significantly different from the catch in white traps (*t* = 0.91, df = 6, *p* < 0.3975).

## Discussion

The results of this study showed that using 100 g of dates per trap was sufficient to achieve good trapping efficacy; however, the catch was not significantly different from that obtained with 300 or 500 g per trap. Given the low quality of dates used in traps, and consequently their low prices, farmers in UAE could afford to use 300 g of dates per trap. It was evident that exceeding 300 g did not significantly improve trap catch. Therefore, from these results, it is recommended to add 100 g of dates per trap for optimum trapping efficacy. Fermented dates emit volatile chemicals that attract adults of *R. ferrugineus*. Faleiro (2005) emphasized the need for using water along with food baits to maintain trap efficacy. The presence of bait volatiles has a synergistic effect with the *R. ferrugineus* pheromone (ferrugineol) placed in the trap (Hallett et al. 1999). In our study, using water with no dates resulted in capturing significantly fewer *R. ferrugineus*, despite the presence of aggregation pheromone. Similarly, in an experiment in which the quantity of a food bait (coconut petiole) ranged from 0 to 500 g per trap, Faleiro (2005) reported that using 200 g per trap was sufficient to maintain good trapping efficacy.

Trap color affects the efficacy of *R. ferrugineus* pheromone traps (Hallett et al. 1999; Ajlan and Abdulsalam 2000; Sansano et al. 2008; Al-Saoud et al. 2010). The results of this study indicated that black was the most attractive color for trapping *R. ferrugineus* adults. The catch in black traps was 1.9 times higher than the catch in white traps. Because white traps are currently used in UAE date palm farms, these higher capture numbers indicate that using black traps could almost double trapping efficacy. From an insect management standpoint, this is a major trapping improvement that is expected to significantly reduce the population size of this major pest in UAE. According to the chromameter color values, it is likely that *R. ferrugineus* adults were attracted to the colors with low *L*-values. In the current study, black was the darkest color, and had the lowest *L-*value. Based on these results, trap surfaces with lower *L*-values should be tested in future studies in order to determine if this can further improve trap attraction. In conclusion, using a black pheromone trap containing 100 g of dates can give better control results and, eventually, will likely contribute to a significant reduction in the population of *R. ferrugineus*. This trap is an excellent tool in the management of *R. ferrugineus* because it is an environmentally sound control device that does not generate chemical pollution, or lead to insecticide resistance problems.

## References

[bibr01] Abraham VA, Al Shuaibi MA, Faleiro JR, Abozuhairah RA, Vidyasagar PSPV (1998). An integrated management approach for red palm weevil, *Rhynchophorus ferrugineus* Oliv. - A key pest of date palm in the Middle East.. *Sultan Qaboos University Journal of Scientific Research, Agricultural Science*.

[bibr02] Abraham VA, Faleiro JR, Kumar TP, Al-Shuaibi MA (1999). Sex ratio of red palm weevil *Rhynchophorus ferrugineus* Olivier captured from date plantation of Saudi Arabia using pheromone traps.. *Indian Journal of Entomology*.

[bibr03] Ajlan AM, Abdulsalam KS (2000). Efficiency of pheromone traps for controlling the red palm weevil *Rhynchophorus ferrugineus* Olivier (Coleoptera: Curculionidae), under Saudi Arabia conditions.. *Bulletin of the Entomological Society of Egypt* (*Economic Series*).

[bibr04] Al-Saoud AH, Al-Deeb MA, Murchie AK (2010). Effect of Color on the Trapping Effectiveness of Red Palm Weevil Pheromone Traps.. *Journal of Entomology*.

[bibr05] El-Ezaby FA, Khalifa O, El Assal A, Al-Afifi MA, Al-Badawi AA (1998). Integrated pest management for the control of red palm weevil in the UAE Eastern Region, Al Ain.. *Proceedings of the First International Conference on Date Palms, 1998*..

[bibr06] Faleiro JR (2000). Investigation of the role of pheromone trapping in the suppression of red palm weevil *Rhynchophourus ferrugineus* Oliv. population in coconut plantations.. *Proceedings of International Conference on Managing Natural Resources for Sustainable**Agricultural Production in the 21^st^ Century*, 2000..

[bibr07] Faleiro JR (2006). A review of the issues and management of the red palm weevil *Rhynchophourus ferrugineus* (Coleoptera: Rhynchophoridae) in coconut and date palm during the last one hundred years.. *International Journal of Tropical Insect Science*.

[bibr08] Faleiro JR (2005). Pheromone technology for the management of red palm weevil *Rhynchophourus ferrugineus* Olivier (Coleoptera: Rhynchophoridae) - a key pest of coconut.. *Technical Bulletin* 4..

[bibr09] Hallett RH, Oehlschlager AC, Borden JH (1999). Pheromone trapping protocols for the Asian palm weevil, *Rhynchophorus ferrugineus* Olivier (Coleoptera: Curculionidae).. *International Journal of Pest Management*.

[bibr10] Murphy ST, Briscoe BR (1999). The red palm weevil as an alien invasive: biology and prospects for biological control as a component of IPM.. *Biocontrol News and Information*.

[bibr11] Salama HS, Saker MM (2002). DNA fingerprints of three different forms of red palm weevil collected from Egyptian date palm orchards.. *Archives of Phytopathology and Plant Protection*.

[bibr12] Sansano Javaloyes MP, Gomez Vives S, Ferry M, Diaz Espejo G (2008). Field trials for the improvement of the effectiveness of the trapping system of the red palm weevil, *Rhynchophorus ferrugineus*, Olivier (Coleoptera: Dryophthoridae).. *Boletin de Sanidad Vegetal, Plagas*.

[bibr13] SAS Institute. (2001). *SAS Users Guide*, Release 8.0 ed..

